# The role of healthcare providers and caregivers in monitoring critically ill children: a qualitative study in a tertiary hospital, southern Malawi

**DOI:** 10.1186/s12913-024-11050-8

**Published:** 2024-05-07

**Authors:** Daniel Mwale, Lucinda Manda-Taylor, Josephine Langton, Alice Likumbo, Michael Boele van Hensbroek, IMPALA Study team, Job Calis, Wendy Janssens, Christopher Pell

**Affiliations:** 1 Kamuzu University of Health Sciences, Blantyre, Malawi; 2 Department of Paediatric Infectious Diseases, Emma Children’s Hospital, Amsterdam UMC, Meibergdreef, NL the Netherlands; 3 Amsterdam Institute for Global Health and Development, Amsterdam, The Netherlands; 4 Training Research Unit of Excellence, Blantyre, Malawi; 5 Department of Economics, Vrije Universiteit Amsterdam, Amsterdam, the Netherlands; 6 Department of Global Health Amsterdam, Amsterdam UMC, location University of Amsterdam, Amsterdam, the Netherlands; 7 Amsterdam Public Health Research Institute, Amsterdam, the Netherlands; 8 Department of Paediatrics and Child Health, Kamuzu University of Health Sciencies, Blantyre, Malawi

**Keywords:** Paediatric critical care, Monitoring, Qualitative research, Malawi

## Abstract

**Background:**

Critically ill children require close monitoring to facilitate timely interventions throughout their hospitalisation. In low- and middle-income countries with a high disease burden, scarce paediatric critical care resources complicates effective monitoring. This study describes the monitoring practices for critically ill children in a paediatric high-dependency unit (HDU) in Malawi and examines factors affecting this vital process.

**Methods:**

A formative qualitative study based on 21 in-depth interviews of healthcare providers (*n* = 12) and caregivers of critically ill children (*n* = 9) in the HDU along with structured observations of the monitoring process. Interviews were transcribed and translated for thematic content analysis.

**Results:**

The monitoring of critically ill children admitted to the HDU was intermittent, using devices and through clinical observations. Healthcare providers prioritised the most critically ill children for more frequent monitoring. The ward layout, power outages, lack of human resources and limited familiarity with available monitoring devices, affected monitoring. Caregivers, who were present throughout admission, were involved informally in monitoring and flagging possible deterioration of their child to the healthcare staff.

**Conclusion:**

Barriers to the monitoring of critically ill children in the HDU were related to ward layout and infrastructure, availability of accurate monitoring devices and limited human resources. Potential interventions include training healthcare providers to prioritise the most critically ill children, allocate and effectively employ available devices, and supporting caregivers to play a more formal role in escalation.

**Supplementary Information:**

The online version contains supplementary material available at 10.1186/s12913-024-11050-8.

## Introduction

For children in low- and middle-income countries (LMICs), trauma and infections, such as sepsis, pneumonia and malaria, are common and contribute to a high burden of critical illnesses [1]. Critical illness is defined as vital organ dysfunction and an increased risk of imminent death, as a result of compromised airway, breathing or circulation [2]. Many deaths due to critical illness are preventable through early diagnosis and timely intervention [2]. Critically ill children require high-quality and essential care starting from the emergency department and in specialised in-patient hospital wards, including high-dependency and paediatric intensive care units (HDUs and PICUs) [1]. In high-income countries (HICs), critical care is often provided with expensive equipment, appropriate laboratory support and specialised personnel [3]. In LMICs, where the childhood disease burden is high and critical care needs are often under-reported, this model is usually impractical because the required resources are either unavailable or not affordable [3, 4].

Vigilant monitoring of children in HDUs or PICUs is a cornerstone of effective critical care, because these patient’s conditions can deteriorate rapidly and life-threatening events may occur [5]. Vital signs, including temperature, blood pressure, pulse rate, respiratory rate and oxygen saturation, provide information about the physiological status [6, 7]. Hence, using vital signs as indications for modifying the acute treatment, is the basis of modern critical care regimen [6]. In addition to clinical observations, vital sign monitoring systems can enable health professionals (doctors, clinicians and nurses) and even accompanying caregivers (mothers, fathers, grandmothers, grandfathers, aunts, uncles etc.) to detect crucial changes on time and potentially save lives [5]. Nonetheless, to achieve high-quality critical care, effective monitoring of relevant (vital) signs must be combined with an appropriate and timely response.

In LMICs, a lack of functional monitoring equipment and qualified human resources makes it challenging to detect and intervene during acute deterioration of critically ill children [8]. Consequently, monitoring of these vulnerable children often relies on ward attendants (hospital domestic workers) and accompanying caregivers. They are not trained in recognising and reporting deterioration, hence contributing to the delays in response [8]. Unfortunately, very little is known about the care and monitoring of critically ill children by healthcare providers and caregivers in HDUs/PICUs in LMICs, as well as how the social, cultural and health system context affects the care provided. With the aim of informing efforts to improve the monitoring of critically ill children in LMICs, this article describes the monitoring practices of healthcare providers and caregivers in a paediatric HDU, and examines the factors that affect the quality of care and monitoring in this setting.

## Methods and setting

### Study design

Using qualitative research methods, this formative study aimed at guiding the implementation and design of an innovative monitor as part of the IMPALA project (“Innovative Monitoring in Paediatrics in Low resource setting: an Aid to save lives”). The findings of this study helped to inform the implementation of the IMPALA study by establishing the current standard of care and healthcare providers’ knowledge on usage of monitors. With support from the European Commission’s EDCTP programme, the IMPALA project aims to develop an affordable, durable, robust and easy-to-use paediatric monitoring system for LMICs. The IMPALA monitor aims to help healthcare providers to track vital sign changes early in the course of disease in under five children admitted in the HDU. The IMPALA vital signs monitor (prototype 2.0) will continuously record vital signs during the admission to these units. Vital signs include heart rate and heart rate variability, respiratory rate, movement, oxygen saturation and non-invasive blood pressure. The study utilised in-depth interviews and structured observations to describe healthcare providers and caregivers’ monitoring of critically ill children. The study was guided by phenomenological theory, whereby the process of collecting data focused on the lived experiences of caregivers and healthcare providers.

### Study setting

**Fig. 1:**
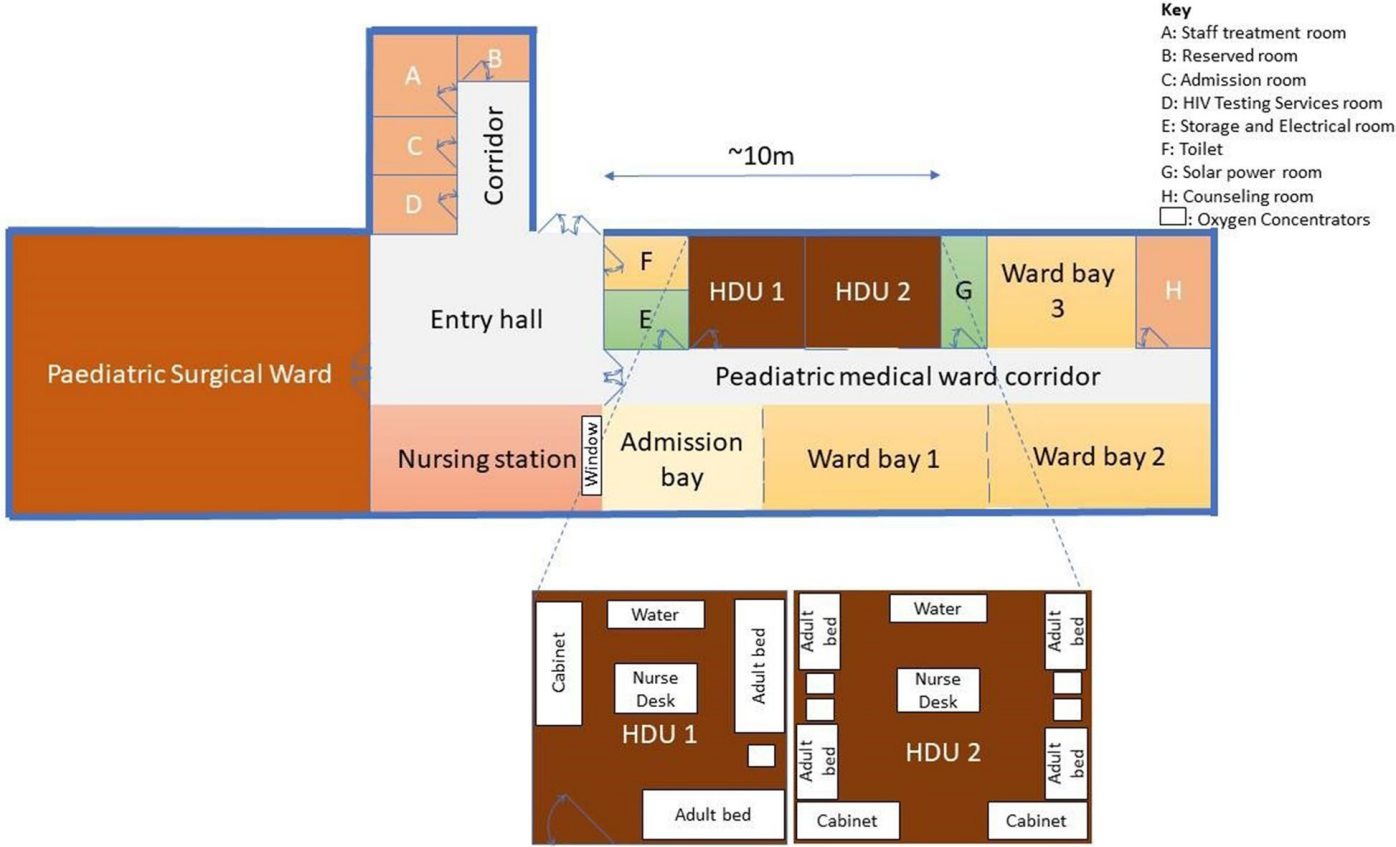
Floor plan of the paediatric ward

The study was conducted in the paediatric HDU of Zomba Central, a tertiary hospital in southern Malawi. Zomba is a large rural district with an estimated population of 746,000 [9]. Zomba Central is a referral hospital for four other hospitals in the region. Hospital policy mandates that all routine care is free to the user [10]. Figure 1 outlines the floorplan of the Zomba Central’s paediatric ward. This includes an admission room where children are assessed and triaged for admission to the medical or surgical wards. The paediatric HDU has eight beds in two separate neighbouring rooms in the ward. The HDU 1 has a door with three adult beds and HDU 2 has five adult beds. This is separated by two movable cabinets from the medical ward. Both HDUs admit critically ill children and those needing extra monitoring, in additional to the routine six hourly monitoring. The triage system for HDU admission follows airway, breathing pattern, circulation and convulsions assessment as well as level of consciousness assessments using the Blantyre coma score. Due to the limited number of beds, two and sometimes three children, regardless of their medical conditions/levels of severity, share the same bed. At least one caregiver is allowed to be present at the admitted child's bedside during the hospitalisation. The age range for HDU admission is below 12 years old, with critically ill neonates admitted at a separate neonatal ward. During the rainy season, an average of around 10 children are admitted each day to the HDUs. The most common reasons for admission include malaria, sepsis and pneumonia. In the dry season, the number of daily admissions declines to around six to eight. The paediatric department has two shifts (day and night). Day (8 h) and night (16 h) shifts have one nurse assigned to the HDUs respectively. A doctor (paediatrician and consultant), intern doctor and a clinician are assigned to the paediatric ward on day and night shifts. Therefore, a total of five nurses and two clinicians are assigned to the paediatric department on day shifts while night shifts consists of four nurses and one clinician (Table 2 describes healthcare provider characteristics). The healthcare providers are rotated on a weekly basis. At the time of data collection, the HDU had two mobile monitors, one pulse oximeter, four oxygen concentrators and one non-functional wall-mounted monitor.

### Sampling and recruitment of study participants

Study participants (healthcare providers and caregivers) were recruited for the structured observations and in-depth interviews between April and June 2022. Study participants were conveniently recruited for the structured observations and in-depth interviews. Healthcare providers of different cadres working in HDU were recruited (face-to-face) during their shifts. Caregivers of critically ill children with a range of diagnosis were also recruited during their admission in HDU. The diversity sample of critically ill children were selected based on the following criteria: age (28 days to 60 months) and categorised as (a) febrile respiratory (e.g., pneumonia, bronchiolitis and asthma), (b) febrile non-respiratory (e.g., malaria and anaemia) or (c) neurological (e.g., head injuries) conditions. Caregivers included anyone accompanying the sick child during his or her hospital stay, typically the mother, aunt or grandmother. The recruitment of participants continued until theoretical saturation i.e., when no new/additional information was forthcoming.

### Data collection

A trained Malawian social scientist with 10 years’of experience in qualitative research (DM), conducted the interviews and observations. In addition, AL (a registered nurse) assisted with data collection, particularly conducting structured observations. Supervision was carried out by LMT, a senior Malawian researcher with a background in qualitative social sciences and bioethics along with 20 years of experience in health-related research. Senior non-Malawian researchers, including a qualitative researcher (CP) and an development economist (WJ) with 15 years and 30 years of experience in health-related research in sub-Saharan Africa, respectively, were also involved. Dutch paediatricians/intensivists (MBvH and JC) and a British paediatrician (JL) and with decades of clinical experience in Malawi, provided additional advice on data collection and analysis.

#### Observations

Two observation guides (healthcare providers and patients) (Additional files 1 and 2) were used to collect data on various areas of critical care in the HDU, including communication between healthcare providers and caregivers during the stay in the HDU, frequency of documenting patient vital signs, the monitors in use, response to alarms, treatments provided and other clinical management based on patient condition. The guides were based on those used for an earlier assessment quality of care in a network of critical care units in diverse LMICs. Patient observations centred on the child during the day shift from the point of HDU admission to either HDU discharge or death. Healthcare providers observations focused on their activities throughout a working shift. The researcher conducted the observations with the support of the research nurse (AL) and systematically recorded the observations using the tool. Furthermore, clarification on the care and treatment provided was sought from healthcare providers, study team and caregivers. However, AL (a registered nurse) could intervene if an emergency arose.

#### Interviews

A multidisciplinary team of social scientists and clinicians developed the interview guides for healthcare providers (Additional file 3) and caregivers (Additional file 4). They included the following topics: experiences during admission, vital signs monitoring processes and responding to acute changes in health. The semi-structured interview guides included open-ended questions, which were adapted during data collection. Interviews were conducted in Chichewa, the local language, to ease the understanding and expression of the participants, with the help of a translator. The interviews were conducted in a private room (nurse’s rest room) or a place selected by participants (within the hospital vicinity) and lasted for thirty to forty five minutes. Caregivers were interviewed on discharge or when the child was stable (during admission). Healthcare providers were interviewed during lunch breaks or at the end of their shifts to avoid disrupting daily tasks. Furthermore, due to their busy schedules, it was not possible to interview healthcare providers at a standardized point in their shifts. To supplement the interviews, observations of their activities were also conducted.

### Data processing and analysis

The researcher recorded in-depth interviews using a digital audio recorder. Audio-recorded data was directly translated from Chichewa to English by an experienced independent transcriber. This data was then reviewed for accuracy by the researcher. Any information identifying the study participants, such as names, was removed from the transcripts. The researcher also kept a field diary with notes on data collection challenges and emerging issues. The audio recordings and complete translated data were stored on a password-protected computer. Microsoft Word documents were imported into QSR NVivo software for organisation, management, and thematic analysis. DM and CP defined terms and concepts from three transcripts, which were used to guide the development of the codebook (Additional file 5) for thematic analysis. The initial codebook was based on initial study objectives and adapted according to emerging issues, with a deductive and inductive approach to coding. Thematic analysis in this case involved identifying, analysing and reporting patterns (themes) within the presented data [11]. The researchers (DM and CP) read each transcript, re-read, and coded the textual data on the participant’s viewpoints and narrative. Subsequently, DM coded the entire dataset based on an agreed coding framework by CP.

### Ethical considerations

The University of Malawi’s College of Medicine Research and Ethics Committee (COMREC) granted ethical approval for the research (P.01/22/3552) on 6th October 2021. All methods followed the Declaration of Helsinki and COMREC guidelines on Health Research. Permission was obtained from the Zomba Central Hospital. Written informed consent was provided by caregivers (mothers/grandmothers/aunt of critically ill children) and healthcare providers with a signature or thumbprint. Participants were informed that confidentiality would be maintained, no personal details would be divulged, their involvement was voluntary and that withdrawal was permitted at any time and without any consequence to their child’s management.

## Results

A total of 21 in-depth interviews were conducted in Zomba Central’s paediatric HDU, with healthcare providers (*n* = 12) and caregivers (*n* = 9) of nine critically ill children (aged 3 months to 5 years old) admitted to the HDU for an average of three days. Structured observations were conducted with the same 12 healthcare providers, 10 caregivers and 10 children. One child died during the observation period and the caregiver was not interviewed because she could not respond to the interview while bereaved and in shock due to the death of the child. The observed children had been diagnosed with conditions including cerebral malaria, severe anaemia, tuberculous meningitis, congenital heart disease, head injury, gastroenteritis and meningitis (Table 1). Over a period of 25 days, a total of 200 h of observations were conducted.

**Table 1:** Observed children admitted to the HDU

Age	Gender	Diagnosis
3 months	Male	Severe pneumonia
7 months	Female	Severe anaemia and severe acute malnutrition
8 months	Male	Meningitis
10 months	Female	Gastroenteritis
1 year.	Female	Congenital heart disease
2 yrs.	Female	Head injury
3 yrs.	Male	Severe anaemia and malaria
3 yrs.	Male	Tuberculous meningitis
4 yrs.	Female	Cerebral malaria and severe anaemia
5 yrs.	Female	Severe malaria

The observed/interviewed healthcare providers were from three cadres (9 nurses, 2 clinical officers, and 1 paediatrician) (Table 2). Characteristics of the study population is summarised in Table 2.

**Table 2:** Interviewed healthcare providers and caregivers

	**Healthcare providers**	**Caregivers**	**Total**
Gender			
Male	3	0	3
Female	9	9	18
Age group			
20–30 yrs.	4	6	10
31–40 yrs.	7	2	9
41–50 + yrs.	1	1	2
Education Level			
None	0	2	2
Primary	0	4	4
Secondary	0	3	3
Diploma	4	0	4
Bachelor’s Degree	7	0	7
Master’s Degree	1	0	1
Occupation			
Housewife		7	7
Farmer		1	1
Businesswoman		1	1
Position			
Nurse Midwife Technician	3		3
Nursing Officer	4		4
Senior Nursing Officer	1		1
Principle Nursing Officer	1		1
Clinicians	2		2
Paediatrician	1		1
Years of experience			
1–9 Months	1		1
1–5 yrs.	4		4
6–10 yrs.	6		6
11–21 + yrs.	1		1

Healthcare providers described vital sign monitoring as crucial in detecting acute changes in the condition of admitted children, in assisting diagnosis and prognosis, and in enhancing the clinical decision-making. They used vital signs thresholds, related to temperature and respiratory rate, which were collected with a thermometer and mobile monitor every six hours, as prescribed by the standard operating procedure, for example, to identify acute changes during admission. The frequency of vital signs checking varied with the severity of illness, with the vital signs of some children taken every hour. However, the analysis of the interviews and observations highlighted how the setting of the HDU created challenges in monitoring the admitted children. These challenges instigated responses from the unit’s healthcare providers and the children’s caregivers. The following section details the main challenges related to the ward layout, monitoring tools, electricity supply and human resources. The response to these challenges encompassed prioritisation of monitoring resources, manual monitoring and the involvement of caregivers.

### Challenges of monitoring

#### Ward layout

All healthcare providers indicated that the separation of the ward from the nursing station hindered the monitoring of admitted children. With the nursing station orientated away from the HDU, and two sets of wooden doors in-between, there was no direct line of sight from the nursing station into HDU1 and HDU2. Hence, neither the admitted children nor the monitors could be seen from the nursing station. There was no central monitoring system in the ward.

*“Yes, I think the ward was not designed well. I think the nursing station was supposed to face the HDUs. There should be a glass door so the nurses can see the HDU from the nursing station. When eating or resting at the nursing station, the HDU is separated. It is difficult for a nurse to know that the child is convulsing unless the caregiver informs you that the child is deteriorating. If not, you only realise the child convulsed when you go to the ward”.* (Nurse IDI 04)

#### Tools

**Fig. 2:**
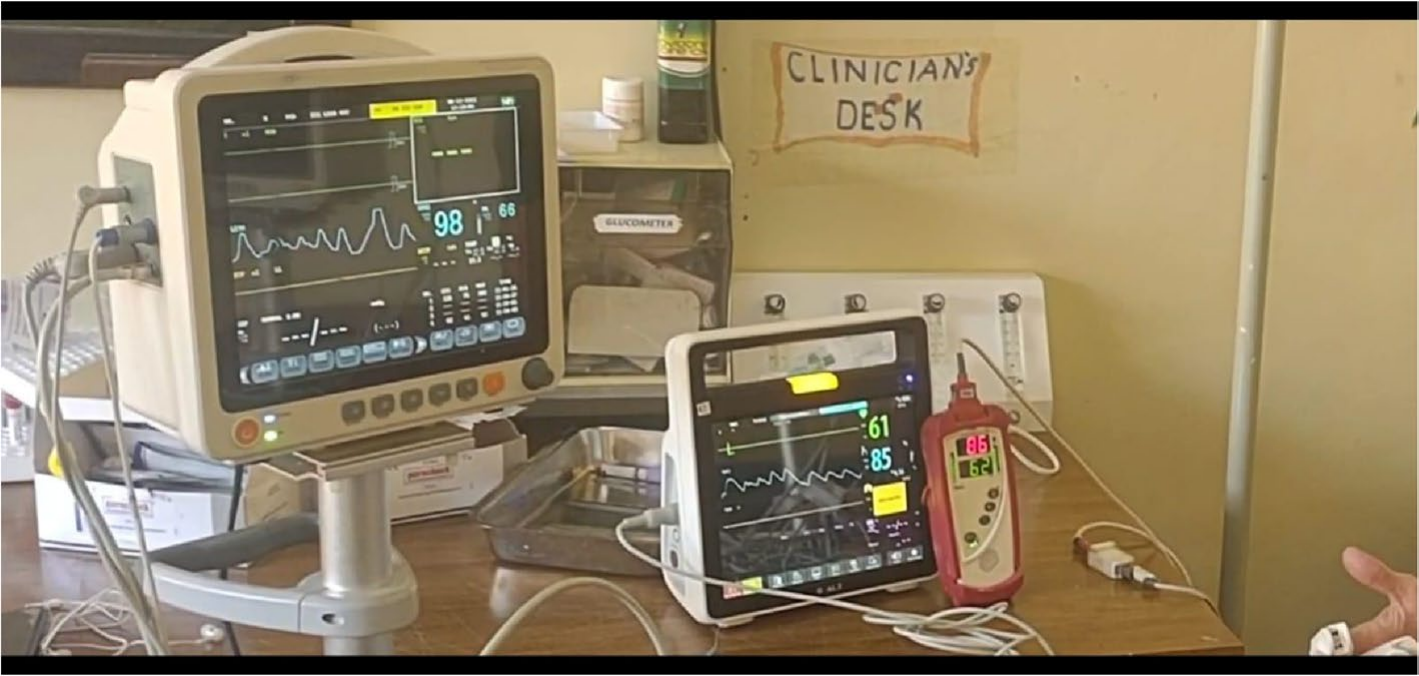
Some of the monitors in use in HDU1 and HDU2

Clinicians and nurses described a lack of adequately functioning equipment, as a crucial challenge in monitoring critically ill children. The monitors and equipments available in the ward comprised of (Fig. 2): a wall-mounted Blocare (PM-900) monitor, two portable multi-parameter vital sign monitors (ARI patient monitor M-9000E) with reusable pulse oximeters, ECG leads, blood pressure cuffs and built-in batteries; a handheld pulse oximeter (Masimo Pronto pulse co-oximeter & haemoglobin analyser) measuring the oxygen saturation and pulse rate (operating on AA batteries); a digital thermometer (BLIP 2) (operating on a coin cell battery); a ZUG medical systems (NP-01NE) sphygmomanometer for measuring the blood pressure (on AA batteries); a SD check gold glucometer (on AA batteries), Pumani bubble CPAP, two NewLife Intensity AirvEP concentrators and one Yuwell 7 F-5 oxygen concentrator.

Healthcare providers expressed concerns that the two portable monitors provided inaccurate results, affecting precise monitoring of critically ill children. They called the monitor “chi 98”, which translates to “always 98”, because the oxygen saturation reading always indicated 98%. Therefore, they preferred using the handheld pulse oximeter.

*“Nurses trust specific machines. For example, the red pulse oximeters are the ones people like the most. […] When the red ones give a reading of 90, it is always 90. We trust them so much because we have used them for a long time.”* (Nurse IDI 03).

#### Electricity supply

All healthcare providers and caregivers indicated that interruptions to the main power supply complicated vital signs monitoring. The interruptions resulted from voltage fluctuations caused by instability in the electricity grid and problems in the solar panels used to supplement the power supply to the paediatric ward. Furthermore, all portable monitors were mainly power-dependent because of worn-out internal batteries and hence could not be used when power was interrupted. Appropriate batteries were often unavailable for pulse oximeters and were too expensive to replace. Other equipment, including oxygen concentrators, was also affected by interruptions to the main power supply.

*“That is a big problem, mainly for paediatric ward nine, where we use solar power. They do not give enough and steady power supply. […] I do not understand why the power keeps on fluctuating. The lights are on and off. The power supply is a problem; it is always there daily. It affects our [care] delivery. We are worried about patients on oxygen therapy, which is a big problem. It becomes problematic if these monitors are in high demand because they rely on the power supply.*” (Clinician IDI 12).

#### Human resources

All healthcare providers mentioned that insufficient staff numbers limited vital sign monitoring and affected the recognition of, and response to, changes in the condition of the admitted children. A lack of trained and qualified healthcare providers in the HDU meant that those present had to manage multiple tasks, such as handling emergencies, prioritisation of critically ill children against the available monitoring equipment, admissions, cannulation and medicine administration, all of which limited their ability to monitor vital signs.

*“As you have observed, we have two nurses in the HDU to look after eight and even more than 12 children. Those critically ill children have different needs; some may need close monitoring, while some may not. This requires us to do many things like admissions, giving medication, conducting ward rounds and resuscitation. We do so many things and we cannot take vital signs the whole time. It is challenging to do it more often because of shortage of staff and resources”.* (Nurse IDI 05)

The healthcare providers described a lack of formal training and saw this as the reason they were unable to use some monitors that eventually led to errors in their use. Preference was given to portable monitors and pulse oximeters, which were considered easy to use, unlike the wall-mounted monitor. The wall-mounted monitor was described as having many probes that required training to use appropriately. Without specific training on how to use these monitors, the healthcare providers mostly relied on practical training, with colleagues who had already been trained, to instruct them. Healthcare providers who had been trained, attended the emergency triage assessment and treatment (ETAT) course, which demonstrated the recognition and treatment of acutely ill children.

*“One is supposed to ask when they are new to anything, so I just asked my colleague and they told me how to use it. This affects me sometimes because you can misinterpret the readings, mainly with the portable monitors.”* (Nurse IDI 09).

### Responding to the challenges of monitoring

#### Prioritisation of monitoring resources

The limited equipment and human resources meant that the routine six hourly and timely monitoring of all admitted patients in the HDU was challenging. Healthcare providers therefore prioritised children for the use of the available monitors. This prioritisation involved the nurses and doctors reflecting and agreeing on the seriousness of a child’s condition based on visual and audible signs related to the patient’s airway, breathing (rising and falling of the chest, breathing sounds and respiratory rate) and circulation (pulse, capillary refill time, body temperature, signs of dehydration such as sunken fontanelle, sunken eyes and skin pinch assessment) during admission, ward rounds and routine assessments. Observations indicated that the most critically ill children for instance those presenting with respiratory and heart conditions (in need of respiratory support) or conditions associated with low levels of consciousness (in need of constant neurological assessment) had their vital signs checked regularly (every thirty minutes or one hour) using portable monitors. In contrast, vital signs for the less critical children (stable children that are not presenting with chest indrawings, constant convulsions or in need of fluid regulation) in the HDU were collected at least every six hours (4 am, 10 am, 4 pm and 10 pm) as required by the standard operating procedures in this HDU. The same monitors were moved from child to child for both non-critically ill children and critically ill children across the whole paediatric ward and not only the HDU to check vital signs. The following vital signs were usually collected: heart rate, respiratory rate, oxygen saturation and temperature.

*“The challenge is that there are two portable monitors and we have 100 patients who may need vital signs checked regularly. We cannot attach a monitor to one patient. They are portable monitors and are used by many patients. […] Ever since I came here, I have never had such a case where we attached a child to a monitor and continuously monitored.” (Nurse IDI 03)*.

“*For those admitted, it could be the ones on the emergency list or priority. The ones in an emergency are the ones we categorise as critically ill. Critical care pathway charts require that we check vital signs regularly, but it depends on my judgement of the patient’s condition because if it is an emergency, I cannot wait for the next four hours to check the vital signs. Therefore, the child’s condition dictates how frequently we check the vital signs. For some, you must do it every fifteen minutes, every hour, and every two hours.” (Nurse IDI 03)*.

#### Clinical observations

Healthcare providers observed breathing patterns and sought to identify signs of dehydration, malnutrition, anaemia and jaundice. Children’s responsiveness (using the Blantyre Coma Score) and capillary refill time were also examined. However, nurses and clinicians indicated that some of the clinical observations could not provide accurate enough information to support conclusive clinical decision-making.

*“If you don’t take the vital signs, you won’t know how exactly the child is. You won’t be able to make the right decision. Yes, you can use your physical observation and judgement because we sometimes judge by appearance. We observe how the children are breathing and the heart rate can be checked manually. You press the artery and set a timer. For temperature, it can also work though it is challenging. It is a challenge, but you can feel the body and you can make the decision. But that will not be the accurate reading.” (Nurse IDI 08)*.

#### The role of caregivers

Caregivers were generally the mothers or grandmothers of the children admitted to the HDU. It was perceived culturally acceptable by healthcare providers and caregivers for women to act as main caregivers and take responsibility for taking care of the children. They were permitted to remain in the unit throughout the child’s hospitalisation and would usually remain near the child, either standing, seated or lying down on the bed. Healthcare providers described the role of caregivers as highly significant in monitoring children admitted to the HDU. Caregivers were observed monitoring children’s status and reporting any noticeable changes in their condition to healthcare providers. For instance, caregivers were observed touching their children to check their body temperature and breathing pattern and talking to them to verify if they were awake and responsive. Furthermore, because they were always at the bedside and were familiar with their children’s conditions prior to falling ill, they could compare changes over time and identify acute changes. It was observed that no formal agreement between the healthcare providers and the caregivers was made to undertake such tasks. Besides monitoring, caregivers had multiple roles, such as holding critically ill children for cannulation and suppository insertions, administering tepid sponging, feeding the children and assisting in oral drug administration on stable children. Through learning from the nurse’s experiences, caregivers were regularly observed reconnecting detached sensors, such as oxygen saturation sensors.

*“I would sit by the bedside so that I could observe my child and whenever he was crying and wanted to remove the [probes], I could put them back.”* (Caregiver IDI 01).

*“I could hold my child’s feet so that the nurses shouldn’t have problems when putting on [equipment for measuring blood oxygen concentration] on the toe. I make sure that the [oxygen tubes] do not fall or be removed. I could also administer drugs like [paracetamol] and report any deterioration to the nurses. I have been doing this several times since I came here. As you know, my child is still young and sometimes can remove the tubes, so I ensure that if it is removed, I take it back.”* (Caregiver IDI 02).

## Discussion

This is the first study to employ qualitative research methods to systematically examine the monitoring practices of healthcare providers and caregivers in a paediatric HDU in sub-Saharan Africa. All healthcare providers recognised the importance of checking vital signs to monitor the condition of the admitted children for timely intervention. However, the study identified barriers to the regular monitoring of children, related to the ward layout, the quality and quantity of available equipment and human resources (numbers of healthcare providers, and the level of critical care training). In the context of these challenges, vital signs were mostly monitored intermittently, using devices that were shared between patients, and clinical observations undertaken. The findings indicate opportunities to improve monitoring, potentially supporting healthcare providers and/or building on the involvement of caregivers.

The layout of the paediatric ward meant that there was no direct line-of-sight from the nursing station to HDU1 or HDU2. Spending more time in the HDU could facilitate closer monitoring of children, yet staff shortages meant that those present were often overburdened with multiple tasks. In many PICUs and HDUs, particularly in high-income countries (HICs), centrally displayed monitoring systems are used to facilitate continuous monitoring from the nursing station [12].

The findings highlight a shortage of reliable monitoring devices that are adapted to their needs and that health providers could use effectively. The available devices assessed a few parameters, such as oxygen saturation and heart rate, simultaneously. Furthermore, the available monitors were malfunctioning, providing inaccurate readings, and could not be easily maintained due to lack of funding. Healthcare providers were not trained to use the available monitors and hence there is also a clear need for training on any additional monitoring devices, particularly as part of on-boarding for new staff members to the HDU (rather than relying on on-the-job training from colleagues).

The shortages in equipment and human resources that were observed in Zomba Central’s paediatric HDU are common to hospitals across the country. For example, a recent survey in Malawi indicated that only 42% of expected hospital staff working days were adequately filled, and 63% of healthcare staff felt they were adequately trained [13]. Moreover, in a recent government audit, in-patient paediatric wards, such as HDUs, mortality was ascribed to inadequate monitoring and delays in instituting emergency treatment [14]. Other research with patients and healthcare providers in Malawi has also highlighted overwhelming staff workload, limited time with clinicians, high patient density in the wards and lack of equipment, such as oxygen and suction machines as significant barriers to providing high-quality care [15]. The healthcare provider shortage results in overburdened clinical staff, overcrowded facilities and limited in-patient monitoring, which may lead to a worsening condition being left under-recognised and hence associated with substantial mortality [16].

In responding to the barriers to continuous monitoring of vital signs, healthcare providers assessed admitted children and prioritised the most critically ill for close and regular monitoring. The available monitors were hence used most frequently for the very critical ill children. The World Health Organisation (WHO) ETAT guidelines are designed as a practical approach to the early assessment and prioritisation for managing critically ill children [17]. In ETAT, several criteria are used: obstructed or absent breathing, severe respiratory distress, central cyanosis, signs of shock (cold extremities with capillary refill time and weak and fast pulse), coma (or seriously reduced level of consciousness), seizures or, in a child with diarrhoea, any two signs of severe dehydration: lethargy or unconsciousness, sunken eyes, very slow return of skin after pinching [18]. The findings indicate that, in a context of limited available tools, high workload and a lack of adequate training on ETAT, healthcare providers apply an informal approach to prioritisation of critically ill children. The ETAT guidelines offer potential for more consistent prioritisation/triage but would require regular training and supportive supervision to ensure their effective implementation. Furthermore, prospective interventions to improve the monitoring of and response to acute changes of critically ill children will require intensive and regular training for healthcare providers to effectively use the available monitors. Moreover, close supervision of the implementation of systematic criteria for the prioritisation of the most critically ill children is essential.

Shortages of healthcare providers, particularly nurses, present a key challenge across the health system in Malawi (and other LMICs), particularly in critical care settings. Hence, caregivers played an important role in monitoring their children in the HDU. Caregivers were observed pointing out their child’s deterioration to nurses, feeding the children and reconnecting the nasal prongs whenever they detached. This echoes the findings of a recent review, which highlighted how, in understaffed hospitals across LMICs, family members often fill the gap by ensuring basic care for their admitted relatives [19]. For example, family members may feed, wash, administer drugs and treat wounds or alert doctors and nurses to clinical deterioration and medical emergencies [20]. However, as was observed in Zomba Central’s HDU, the family caregiver role is generally informal, unsupported and untrained. In settings where healthcare providers face unmanageable workloads, caregivers’ contribution to critical is likely to remain crucial, however, outlining clearly their role and responsibilities is likely to be beneficial [21].

A feasibility study conducted in Kenya suggested that empowering caregivers to assist with the timely recognition of deterioration in hospitalised children might expedite the clinical response and improve health outcomes [22]. With counselling and basic training from healthcare providers, caregivers could better monitor the progress of their hospitalised child, communicate more effectively and indicate concerns in a timely manner [22]. Supporting and training family members to participate in basic care in resource-limited hospitals could provide a safe and adequate opportunity to enhance care while tackling health provider shortages [23]. Additionally, family-delivered care could stimulate quicker recovery, fewer adverse events and better outcomes [24]. Family participation in HICs hospitals has enhanced staff-delivered care, improved emotional support, accelerated recovery and improved discharge preparation [25, 26]. However, the over-reliance on caregivers to recognise and report deterioration can negatively affect patient care. With limited medical knowledge and experience, caregivers may delay in reporting changes to healthcare providers in case of respiratory distress or convulsions. The monitoring of critically ill children should be considered a responsibility shared between healthcare providers and caregivers. Caregivers’ role could focus on escalating critical events to the healthcare providers and making explicit the expectations of the caregivers in terms of monitoring is very crucial. However, interpreting vital signs remains the responsibility of the healthcare providers.

### Strengths and limitations of the study

The study was strengthened by conducting observations of practices in the HDU supplement in-depth interviews with a diverse group of respondents, including healthcare providers and caregivers. Furthermore, the study was undertaken by a team of researchers from the social sciences, paediatric and intensive care medicine, with backgrounds in Malawi and HICs, and data collected by staff with backgrounds in clinical medicine and social sciences. Another strength was observing the care provided to children with varied diagnoses to maximise the applicability of the findings across the children admitted to the HDU. To ensure the reliability of the findings and reduce the potential for the Hawthorn effect (individual behaviour modified in response to their awareness of being observed) and desirability bias, prior to and during data collection, efforts were made to familiarise the healthcare providers with the study aims and the staff who would be involved in data collection by being present over extended periods of time [27]. The study is limited by the data collection period (April and June 2022) and data were collected only during day shifts; paediatric critical care services were hence not observed during periods of higher/lower or different disease burden. In addition, data on clinical outcomes of the observed children were not collected to evaluate the impact of monitoring on clinical outcomes.

## Conclusion

In Zomba Central HDU, all healthcare providers and caregivers recognised the importance of vital sign monitoring among admitted critically ill children for timely intervention. However, healthcare providers faced challenges to monitoring, related to the ward layout, the quality and quantity of available equipment and limited human resources (insufficient numbers of healthcare providers and training). In response, healthcare providers made use of an informal prioritisation system and selected the most critically ill children for close and frequent monitoring. Monitoring was mostly performed by intermittent vital sign checks, using devices shared between patients, and supplemented by clinical observations. Caregivers also monitored their children and communicated acute changes to healthcare providers. Potential interventions to improve the monitoring of and response to acute changes of critically ill children include: training healthcare providers to effectively use the available monitors; supervising the implementation of systematic criteria for the prioritisation of the most critically ill children; and making explicit the expectations of caregivers in terms of monitoring.

### Supplementary Information

**Supplementary Material 1.**

**Supplementary Material 2.**

**Supplementary Material 3.**

**Supplementary Material 4.**

**Supplementary Material 5.**

## Data Availability

The data and materials supporting this study’s findings are available on request from the corresponding author, [DM]. The data are not publicly available due to the privacy and confidentiality of the research participants.
