# Supplementary material for: The role of healthcare providers and caregivers in monitoring critically ill children: a qualitative study in a tertiary hospital, southern Malawi
Source: BMC Health Serv Res. 2024 May 7;24:595. doi: 10.1186/s12913-024-11050-8 (PMC11077805; doi:10.1186/s12913-024-11050-8)
Supplement: Supplementary file 1 — Supplementary Material 1. [file 12913_2024_11050_MOESM1_ESM.docx]

Annex 1: Observation guide: case study staff member (observed over 1 shift)

**Instructions:**

- Find a comfortable place where you do not influence care processes in the hospital

- Obtain verbal informed consent (or assent) from everyone present in HDU

- Fill in the basic details of the observation (time/duration/location etc.)

- Add your notes on the topics below as soon as possible after observing

- Record observations of events and informal conversations

| **Date** |  | **Start time** |  | **End time** |  |
| --- | --- | --- | --- | --- | --- |
|  |  |  | | | **Number** |
| Staff | Nurses | Day shift | | |  |
|  |  | Night shift | | |  |
|  | Doctors | Day shift | | |  |
|  |  | Night shift | | |  |
| Patients | At ICU/HDU at 8am | | | |  |
|  | Admissions in the previous 24 hours | | | |  |
|  | Deaths in the previous 24 hours | | | |  |
|  | Discharges to other wards | | | |  |
|  | Discharges home to die | | | |  |
|  | Discharges home for recovered patients | | | |  |
|  | Transferred to another facility | | | |  |

|  | **Duration in minutes** |
| --- | --- |
| Nursing and doctors’ handover (morning) |  |

|  | **Staff details** |
| --- | --- |
| Job title |  |
| Gender |  |
| Years of experience |  |
| Years at ward |  |

| **Time period** | | **Activities** | **Interruptions / distractions** |
| --- | --- | --- | --- |
| 07:00 | 07:30 |  |  |
| 08:00 | 08:30 |  |  |
| 09:00 | 09:30 |  |  |
| 10:00 | 10:30 |  |  |
| 11:00 | 11:30 |  |  |
| 12:00 | 12:30 |  |  |
| 13:00 | 13:30 |  |  |
| 14:00 | 14:30 |  |  |
| 15:00 | 15:30 |  |  |
| 16:00 | 16:30 |  |  |
| 17:00 | 17:30 |  |  |
| 18:00 | 18:30 |  |  |
| 19:00 | 19:30 |  |  |
| 20:00 | 20:30 |  |  |
