# Supplementary material for: The role of healthcare providers and caregivers in monitoring critically ill children: a qualitative study in a tertiary hospital, southern Malawi
Source: BMC Health Serv Res. 2024 May 7;24:595. doi: 10.1186/s12913-024-11050-8 (PMC11077805; doi:10.1186/s12913-024-11050-8)
Supplement: Supplementary file 2 — Supplementary Material 2. [file 12913_2024_11050_MOESM2_ESM.docx]

**Annex 2**: Observation guide: case study patient (observed over the period of admission)

**Instructions:**

- Find a comfortable place where you do not influence care processes in the hospital

- Obtain verbal informed consent (or assent) from everyone present in HDU

- Fill in the basic details of the observation (time/duration/location etc.)

- Add your notes on the topics below as soon as possible after observing

- Record observations of events and informal conversations

| **Date** |  | **Start time** |  | **End time** |  |
| --- | --- | --- | --- | --- | --- |
|  |  |  | | | **Number** |
| Staff | Nurses | Day shift | | |  |
|  |  | Night shift | | |  |
|  | Doctors | Day shift | | |  |
|  |  | Night shift | | |  |
| Patients | At ICU/HDU at 8am | | | |  |
|  | Admissions in the previous 24 hours | | | |  |
|  | Deaths in the previous 24 hours | | | |  |
|  | Discharges to other wards | | | |  |
|  | Discharges home to die | | | |  |
|  | Discharges home for recovered patients | | | |  |
|  | Transferred to another facility | | | |  |

|  | **Duration in minutes** |
| --- | --- |
| Nursing and doctors’ handover (morning) |  |

|  | **Patient details** |
| --- | --- |
| Age |  |
| Gender |  |
| Diagnosis |  |
| Prognosis |  |
| Date admitted |  |
| Care giver present |  |

| **Patient monitoring** | **Frequency of document per 8 hours it taken manually** |
| --- | --- |
| Blood pressure |  |
| Heart rate |  |
| Respiratory rate |  |
| Oxygen saturations |  |
| FiO 2 |  |
| GCS |  |
| Daily fluid balance |  |

| **Detailed description** | | | | | | |
| --- | --- | --- | --- | --- | --- | --- |
| **Monitors** | Monitors in use  Alarms sounding  Thresholds set  Screens/data visible | | |  | | |
| **Alarms** | Time sounds |  |  | | Time response |  |
|  | Time sounds |  |  | | Time response |  |
|  | Time sounds |  |  | | Time response |  |
|  | Time sounds |  |  | | Time response |  |
| **Response to perturbations** | Who responds to alarms or other indications of deterioration?  Decision-making process | | |  | | |
| **Relatives** | Who is present?  Monitoring role  Communication with health staff | | |  | | |
| **Care** | What treatment has the child received? What tests of examinations? | | |  | | |
| **Communication** | Have staff informed the care giver about the condition of the child?  Have they asked for the care givers opinion? | | |  | | |
| **IMPALA** | Was the IMPALA monitor used?  Any problem?  Did alarms sound? Did staff respond to the alarm?  Comments about IMPALA | | |  | | |
| ***Other points*** |  | | | | | |
|  |  |  |  |  |  |  |
