# Supplementary material for: The role of healthcare providers and caregivers in monitoring critically ill children: a qualitative study in a tertiary hospital, southern Malawi
Source: BMC Health Serv Res. 2024 May 7;24:595. doi: 10.1186/s12913-024-11050-8 (PMC11077805; doi:10.1186/s12913-024-11050-8)
Supplement: Supplementary file 3 — Supplementary Material 3. [file 12913_2024_11050_MOESM3_ESM.docx]

**Annex 3: Healthcare Providers Interview Guide in English**

| **Instructions:**  - Follow the **informed consent** procedures  - If consent is given, **audio record the interview**  - This interview guide is to be used in a **flexible manner**.  - The aim is to collect **in-depth information** from the respondent.  - The left-hand column lists the determinants that influence the implementation of the intervention  - The middle column explains the determinant  - The right-hand column contains a list of suggested questions and probes. It also contains some tick-boxes that you can use to summarize the responses  - It is not necessary to ask all these questions in the order listed; these provide ideas to prompt the respondent to talk about the topic of interest  - Use a flexible approach and probe as necessary: add extra questions depending on the responses you hear.  - You do not need to follow the order the topics as presented below; follow the responses and the flow of the conversation.  - Above, all show interest in the respondent and the answers that he or she gives |
| --- |

| **Topic** | **Suggested questions** |
| --- | --- |
| **Background professional information** | - What is your current role (what are your tasks and responsibilities)?  - How long have you worked in this role?  - What is your age?  - Why did you decide to become a …?  - Where did you work in the past?  - What were your previous roles?  - What professional training have you done in the past? |
| **Vital sign monitoring** | - How do you monitor the vital signs of a children who is admitted?  - What vital signs do you monitor?  - How often do you take vital signs?  - How do you record them?  - Does someone help you take vital signs?  - Are the vital signs used in hand-over?  - Why do you take those vital signs? Why do you take that with that frequency?  - Would you like to take more of them more often?  - Do you monitor some children more than others? Why?  - Which conditions/diagnosis require particular monitoring?  What do you see as abnormal vital signs?  Is this the same for all patients?  What do you do when the vital signs are abnormal?  Do you feel confident in interpreting the vital signs?  (How) do they help in clinical decision making? |
| **Monitors** | - Do you have any monitors that can continuously monitor vital signs?  - Do you use the monitors? If not, why not?  Do you feel confident using the monitors?  Did you ever receive training for using the monitors?  - Do you have any problems with the monitors?  - Do they make alarms?  - Do power cuts cause problems? Do you have problems with setting the monitors? E.g. with setting threshold for children?  - How many types of monitors are in the ward?  - Are any of them broken?  - Who maintains the monitors?  - Do you have the probes you need for monitors?  - How do you decide which child should be attached to a monitor?  Who decides?  When is a child taken off the monitor? For what reasons? |
| **Identifying deterioration** | - Apart from vital signs, what other ways do you monitor the children?  - How do you know if someone is getting more sick?  - Do you get help from the parents/guardians?  - How do they help?  - Do you get help from other staff? Who also monitors the children? How do they let you know if someone is getting sicker?  - What happens in the night or at the weekend?  - What other information would you like on the children’s conditions? |
| **Responding to deterioration** | - What happens when a child gets more sick?  - Who responds first?  - What happens if the alarm on a monitor sounds?  - Who do you contact if you think a child is getting sicker?  - When do you contact a physician or the clinical officer? |
| **Workflow and workload** | - How many patients do you normally attend to in one day? How do you prioritise which patients to treat?  - What tasks take most of your time?  What tasks could be organized more efficiently?  What tasks are most cumbersome?  Do you feel you can complete your tasks in time?  - Which tasks do you wish you could dedicate more time to?  - What prevents you from spending the time you need on those tasks? |
| **Team work and hand-over** | - How often do you talk about the status of patients with your colleagues? What do you discuss?  - How long does handover at the end of shifts take? How do you handover to the next shift?  - How often do you talk to the physicians or clinical officers?  - Are you ever short of staff? Which staff are missing? How often?  - What impact do staff shortages have on monitoring patients or reacting when they are getting sicker? |
| **The ward** | - Are you able to monitor patients from the nursing station?  - When do you stay in the nursing station?  - Do you stay there in the night?  - What tasks do you do there?  - What problems do you have with the way that ward is organized? And where the HDUs are?  - How could the design of the ward be improved? |
| **The hospital** | - Are there any problems in the hospital that hinder the monitoring of patients (e.g. power cuts, lack of staff or equipment)?  - Is there anything in the hospital that prevents you from reacting when a child gets sicker? Can you describe that? |
| **The care for a specific patient** | - Do you remember patient…(describe a particular patient that you observed)  - What was the diagnosis?  - How was the diagnosis made?  - Who made the decision to admit to HDU?  - How did you monitor that child?  - How often did you discuss his/her condition with the parent/guardian?  - Who else was involved in monitoring his/her condition?  - What happened to him/her?  - What treatment did s/he receive?  - Why did s/he receive those treatment?  - Did you discuss the case with other staff?  - Why do feel that s/he got better or worse?  - Could the care have been any better? What could have been improved? |
| **Closing** | - (Give a few sentences about the main points that you have discussed_  - Do you have any questions for me?  - If you’d like to talk more about any of these topics, please contact me.  - Thanks for taking the time to speak to me |
