# Supplementary material for: The role of healthcare providers and caregivers in monitoring critically ill children: a qualitative study in a tertiary hospital, southern Malawi
Source: BMC Health Serv Res. 2024 May 7;24:595. doi: 10.1186/s12913-024-11050-8 (PMC11077805; doi:10.1186/s12913-024-11050-8)
Supplement: Supplementary file 4 — Supplementary Material 4. [file 12913_2024_11050_MOESM4_ESM.docx]

**Annex 4: Caregivers Interview Guide in English**

| **Instructions:**  - Follow the **informed consent** procedures  - If consent is given, **audio record the interview**  - This interview guide is to be used in a **flexible manner**.  - The aim is to collect **in-depth information** from the respondent.  - The left-hand column lists the determinants that influence the implementation of the intervention  - The middle column explains the determinant  - The right-hand column contains a list of suggested questions and probes. It also contains some tick-boxes that you can use to summarize the responses  - It is not necessary to ask all these questions in the order listed; these provide ideas to prompt the respondent to talk about the topic of interest  - Use a flexible approach and probe as necessary: add extra questions depending on the responses you hear.  - You do not need to follow the order the topics as presented below; follow the responses and the flow of the conversation.  - Above, all show interest in the respondent and the answers that he or she gives |
| --- |

| **Topic** | **Suggested questions** |
| --- | --- |
| **Background information** | - How old are you?  - What level of schooling do you have?  - How many children do you have?  - Have you brought another child to paediatrics before? |
| **Care seeking** | - How is your child?  - Can you tell me what is wrong with your child?  - When did you notice that your child was sick?  - Why did you think that he/r was sick?  - What were the symptoms?  - Did you ask anyone else for an opinion about the child’s illness?  - Where did you first seek help?  - Why did you come to the hospital? |
| **Admission** | - When was your child admitted?  - What happened on admission?  - Who did you talk to first?  - What were you told on admission?  - Did you child come straight to the HDU?  - Did anyone explain why s/he was brought to HDU? |
| **Monitoring** | - How has the condition of your child been monitored in the HDU?  - Have you seen any monitors used?  - Who has monitored the condition?  - Have you provided any assistance with monitoring?  - How did you help?  - How could you tell if the condition of your child was getting worse?  - How did other people see that the condition of your child was worsening? |
| **IMPALA (only for 2.0 and 3.0)** | - Did anyone explain to you about the IMPALA monitor?  - What did you hear?  - Did you notice the probe attached to the finger? What did you feel about that?  - Did anyone explain what the device was doing? What were you told?  - Did you notice any alarms that the device made? What did you think about that?  - What happened if the alarms sounded?  - What did you think about the screen on the device? And the images that were shown? |
| **Responding to deterioration** | - Did your child’s condition deteriorate when admitted?  - What happened?  - How did the staff respond?  - Who came to help?  - Did they move the child elsewhere?  - Did they give any extra medicine or do any new procedure? |
| **Information from staff** | - Did staff keep you informed of your child’s care and treatment with nurses or doctors?  - Who and how often did they talk to you?  - Did they staff clearly informed you about the consequences of the treatment?  - What were told about any examinations and tests?  - What were you told about the drugs your child received?  - Did they tell you when your child was getting sicker or better?  - What did they say? |
| **Interactions** | - Did the staff ask for your input into the care that they were providing?  - Did they ask about what you wanted for your child?  - Did they ask your opinion of particular treatments or tests?  - Did they ask you if you were happy for your child to be monitored?  - Did they ask you  - Did staff ask for your thoughts about whether your child was getting better or deteriorating?  - Did they staff talk to you in a respectful manner? Were you happy with they way they talked to you? |
| **The ward** | - Did you ever have to go look for staff to seek their help? What happened?  - Did you approach the nursing station for help or go elsewhere?  - Where have you been sleeping whilst your child is admitted?  - What do you think about the ward? Whether it is clean? Too busy? The spaces for sleeping? |
| **The hospital** | - What do you see as the main problems in the hospital?  - What do you like about this hospital? What do you dislike?  - Were you able to get food whilst your child was admitted? What about drinks? |
| **Overall experience of care** | - What did you think about the way your child was treated when admitted?  - What were you happy and unhappy about?  - Would you come back to this hospital if your child was ill again? Why/why not? |
| **Closing** | - (Give a few sentences about the main points that you have discussed_  - Do you have any questions for me?  - If you’d like to talk more about any of these topics, please contact me.  - Thanks for taking the time to speak to me |
