# Supplementary figures and images for: The role of healthcare providers and caregivers in monitoring critically ill children: a qualitative study in a tertiary hospital, southern Malawi
Source: BMC Health Serv Res. 2024 May 7;24:595. doi: 10.1186/s12913-024-11050-8 (PMC11077805; doi:10.1186/s12913-024-11050-8)

**Annex 5: Coding tree**

**
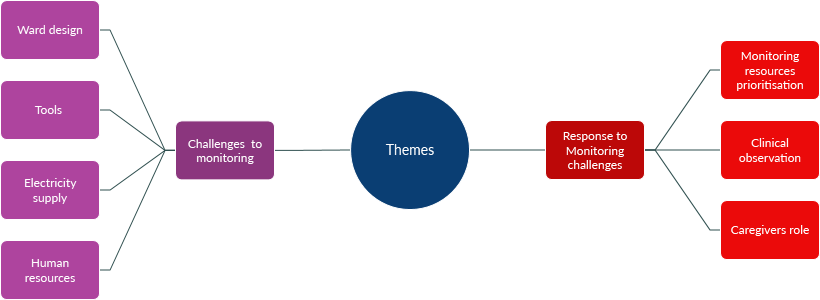
**

Supplement: Supplementary file 5 — Supplementary Material 5. [file 12913_2024_11050_MOESM5_ESM.docx]
